# Factors associated with the preoperative electrophysiological severity of carpal tunnel syndrome and correlation with early postoperative hand function status

**DOI:** 10.3389/fneur.2026.1817141

**Published:** 2026-10-28

**Authors:** Liangyin Zhang, Bing Wang, Yan Su, Shouxi Li, Li He, Qingming An

**Affiliations:** 1 Microsurgery of the Hand and Foot, The First People's Hospital of Baiyin, Baiyin, Gansu, China; 2 Department of Electrophysiology, The First People's Hospital of Baiyin, Baiyin, Gansu, China

**Keywords:** anthropometry, carpal tunnel syndrome, electromyography, electrophysiology, risk factors

## Abstract

**Objective:**

To investigate factors associated with preoperative electrophysiological severity in carpal tunnel syndrome (CTS) and the correlation between electrophysiological severity and early postoperative hand function status.

**Methods:**

A total of 148 patients with CTS diagnosed by clinical examination, nerve conduction studies and supplementary electromyography in the study hospital between 1 January 2024 and 1 December 2024 were retrospectively enrolled in this study. Based on preoperative electrophysiological severity, CTS patients were split into mild, moderate, and severe groups. At 3 weeks post-treatment, we collected demographic data, anthropometric measurements, and scores from the LCTQ, PRWE, and DASH questionnaires. Statistical analyses included one-way ANOVA, chi-square tests, and ordinal logistic regression.

**Results:**

With the escalation of disease severity, disease duration (14.66 ± 5.59 vs. 27.35 ± 11.67 vs. 34.81 ± 11.22, *p* < 0.001) and transverse carpal diameter (36.68 ± 3.03 vs. 38.78 ± 2.76 vs. 40.92 ± 2.89, *p* < 0.001) increased progressively, whereas the ratio of wrist circumference to transverse carpal diameter decreased (4.24 ± 0.39 vs. 4.07 ± 0.39 vs. 3.92 ± 0.38, *p* < 0.001). The ordinal logistic regression showed that disease duration, transverse carpal diameter and wrist circumference/transverse carpal diameter were independent factors associated with electrophysiological severity (omnibus test: *p* < 0.001, Nagelkerke pseudo *R*^2^ = 0.622). Preoperative LCTQ, PRWE and DASH scores were significantly higher in the severe group than in the mild or moderate groups (all *p* < 0.001). At 3 weeks postoperatively, all scores were significantly improved from baseline (all *p* < 0.001) but still differed across groups. Correlation analysis revealed that LCTQ (*r* = 0.63), PRWE (*r* = 0.42) and DASH (*r* = 0.87) results were all positively correlated with preoperative electrophysiological severity.

**Conclusion:**

Disease duration, transverse carpal diameter and the ratio of wrist circumference to transverse carpal diameter were associated with electrophysiological severity in patients with CTS, with longer disease durations, larger transverse carpal diameters and smaller wrist circumference-to-transverse carpal diameter ratios corresponding to greater electrophysiological severity and poorer early postoperative function statuses.

## 1 Introduction

Carpal tunnel syndrome (CTS) is the most common peripheral neuropathy, accounting for 90% of all peripheral neuropathies, with an incidence rate of 1.5–3.8% and a mean age of onset ranging from 40 to 60 years. The majority of patients with CTS exhibit bilateral hand involvement (1). The condition tends to affect women in greater proportion, with a prevalence of 3.0% in women versus 2.1% in men (2, 3). Carpal tunnel syndrome is more commonly observed in populations engaged in occupations involving repetitive hand movements (e.g., housewives, teachers and typists). Its pathophysiological mechanism is that repetitive wrist movements cause a thickening of the transverse carpal ligament and reduction in carpal tunnel volume, which results in the compression of the median nerve between the flexor tendons and the transverse carpal ligament. This compression impairs the microcirculation around the median nerve, resulting in an obstruction of lymphatic and venous return and dysfunction of the axoplasmic flow in axons that lead to decreased permeability of the perineurium and endoneurium; subsequent oedema, degeneration and necrosis of neuroglial cells; and, ultimately, demyelination of the nerve. Demyelinating changes are the predominant pathological feature of CTS in most patients, with axonal degeneration occurring in severe cases (4).

The pathogenesis of primary CTS is currently attributed to the ‘local wrist inflammation theory’, which posits that inflammation induces local swelling and the subsequent compression of the median nerve. Simultaneously, the activation of inflammatory factors such as interleukin-6 leads to local proliferation, resulting in a thickening of the flexor and transverse carpal ligaments and further exacerbation of local compression. The clinical manifestations of CTS include numbness and pain in the wrist and the radial three and a half fingers, wrist weakness, clumsiness of hand movements and difficulty in flexion. Severe cases may present with hand muscle atrophy (2, 5). Carpal tunnel syndrome impairs patients’ grip strength and fine motor skills, exerts adverse effects on their daily activities and social productivity, and may even cause permanent hand disability (2, 6). Therefore, early detection, diagnosis and treatment are crucial to the prognosis of patients with CTS (2, 7).

The diagnosis of CTS is primarily based on patients’ medical history, clinical manifestations and physical examinations. Nerve conduction studies (NCSs) are the gold standard for CTS diagnosis, with needle electromyography (EMG) serving as a supplementary examination (8). Nerve conduction studies in patients with CTS may show prolonged sensory latency of the median nerve or prolonged palmar mixed nerve latency along with reduced sensory conduction amplitude. With disease progression, the distal sensory and motor nerve potential latencies of the median nerve are both prolonged. In patients with severe CTS, both the motor and sensory latencies of the median nerve are prolonged, and this is accompanied by a decreased or absent amplitude. For patients who do not respond adequately to conservative treatments, such as splinting, corticosteroid injections and oral anti-inflammatory medications, surgical decompression of the carpal tunnel is considered the standard therapeutic option. The primary surgical procedure, known as carpal tunnel release, aims to relieve pressure on the median nerve by dividing the transverse carpal ligament. This can be performed via open or endoscopic techniques, both of which have been shown to effectively improve nerve conduction parameters and clinical symptoms. Current clinical guidelines recommend surgical intervention for patients with severe electrophysiological impairment, persistent symptoms despite conservative management or progressive motor deficits such as thenar muscle atrophy. Moreover, increasing electrophysiological severity is generally regarded as a strong predictor of surgical necessity and poorer postoperative function status if treatment is delayed.

Previous studies have indicated that risk factors associated with the onset of CTS include sex, age, occupation, the presence of metabolic abnormalities or rheumatologic immune system diseases, a history of hand trauma and cervical spondylosis comorbidity (9–11). A large body of research has shown that anthropometric measurements exert an effect on the results of NCSs in CTS, yet their correlation with CTS severity remains unclear (7, 12). This study aims to retrospectively analyze and summarize the factors associated with electrophysiological severity in CTS and thereby provide an evidence base for clinical practice and a reference for predicting disease severity, guiding treatment and evaluating prognoses in patients with CTS.

## 2 Materials and methods

### 2.1 Study participants

A total of 148 patients with CTS diagnosed by clinical assessment (13), NCS and supplementary EMG (14) in the study hospital between 1 January and 1 December 2024 were retrospectively enrolled using a convenience sampling method. The inclusion criteria were as follows: patients (1) >18 years of age; (2) conforming to the clinical and electrophysiological diagnostic criteria for CTS; (3) and who voluntarily signed an informed consent form. The exclusion criteria were as follows: (1) patients with autoimmune diseases, elevated inflammatory markers or fever; (2) those with poor skin condition with severe lesions; (3) patients with a history of wrist or forearm fractures or with CTS complicated by advanced tumors, skeletal tuberculosis, severe osteoporosis, wrist cysts or benign tumors; (4) pregnant and lactating women; and (5) patients with abnormal hematopoietic function, coagulation dysfunction or bleeding tendency. During the study period, all patients who underwent electrodiagnostic studies for suspected CTS and met the clinical and electrophysiological diagnostic criteria for CTS were identified through the hospital’s electronic medical record system. Eligible patients were consecutively screened, and those meeting the inclusion criteria and with complete baseline and follow-up data were included in the study. Patients were excluded only if they met any of the predefined exclusion criteria listed above. Therefore, the study population represents a consecutive series of eligible cases rather than a selective subset based on data availability. It should be clarified that although this study followed a retrospective design, all data were collected through standardized, pre-specified protocols as part of our department’s routine clinical workflow for CTS management, rather than *ad hoc* data extraction. For all patients diagnosed with CTS during the study period, demographic information, anthropometric measurements (including transverse carpal diameter measured by digital caliper and wrist circumference measured by flexible tape), electrophysiological assessments, and postoperative functional evaluations were routinely documented in a structured electronic medical record template aligned with our institutional clinical pathway. This standardized documentation practice, implemented prior to study initiation, explains the systematic nature of the collected data and ensures consistency across all enrolled cases.

This study was approved by the ethics committee of the study hospital.

### 2.2 Study method

Patients were diagnosed if they met two or more of the following clinical diagnostic criteria for CTS (13): (1) abnormal sensory symptoms in the affected hand, including numbness, swelling, a sensation of tightness, paraesthesia and pain, mainly distributed in the thumb, the index and middle fingers and the radial half of the ring finger; (2) obvious nocturnal paraesthesia, accompanied by weakness of the palm or fingers in some patients; (3) a positive Phalen’s sign; (4) a positive Tinel’s sign; and (5) abnormal wrist sensations, such as pain, sometimes radiating to the shoulder.

Diagnosis based on the electrophysiological diagnostic criteria for CTS (14) was defined as conformance to the electrophysiological characteristics of CTS formulated in 2011 by the American Association of Neuromuscular and Electrodiagnostic Medicine, with specific manifestations potentially including (1) prolonged or unelicited distal sensory latency of the median nerve, (2) decreased or unelicited amplitude and conduction velocity of the sensory potentials of the median nerve, (3) prolonged motor potential latency of the median nerve, (4) prolonged or absent motor potential latency of the median nerve, (5) decreased or unelicited amplitude and conduction velocity of the motor potentials of the median nerve and, in some cases, (6) the presence of abnormalities on needle EMG. In this study, electrophysiological evaluation included NCSs as the main diagnostic modality, with needle EMG performed for supplementary assessment.

For EMG acquisition, the temperature of the examination room was maintained at 25.0 °C, patients’ skin temperatures were kept above 32.0 °C and patients were placed in a supine position. Nerve conduction studies and needle EMG were performed using an eight-channel Keypoint. NET EMG and evoked potential system (Dantec Medical, Skovlunde, Denmark) (15, 16).

Nerve conduction studies included motor and sensory conduction studies and needle EMG. (1) Motor conduction studies were conducted as follows: the recording electrode (electrode 1 [E1]) was placed on the belly of the abductor pollicis brevis muscle, the reference electrode (electrode 2 [E2]) was placed on the proximal phalanx of the thumb and the ground electrode was placed on the dorsum of the hand. Electrical stimulation was delivered to the median nerve at the wrist (with the cathode of the stimulator placed approximately 6.5 cm proximal to E1) and elbow (with the cathode placed lateral to the brachial artery in the cubital fossa) at the corresponding body surface projection sites of the main trunk to obtain the distal motor latency (DML) at the wrist, the elbow latency and the compound muscle action potential (CMAP) of the median nerve. Conduction time was defined as the difference in latency between the two stimulation sites. The distance between the two sites was measured, and the motor nerve conduction velocity of the median nerve was automatically calculated by the software. (2) Sensory conduction studies were conducted as follows: the antidromic method was adopted for testing, with the recording electrode (E1) placed on the middle finger, the reference electrode (E2) placed approximately 2–3 cm distal to E1 and the ground electrode placed on the dorsum of the hand. Electrical stimulation was applied to the median nerve at the wrist (approximately 13 cm from E1) at the corresponding body surface projection site of its main trunk to obtain the sensory nerve action potential (SNAP) of the median nerve. The distance from the stimulation site to E1 was measured, and the sensory nerve conduction velocity of the median nerve was automatically calculated by the software. (3) Needle EMG was performed as follows: the abductor pollicis brevis muscle was examined routinely, and, based on the patient’s condition, two additional proximal muscles innervated by the median nerve (e.g., the flexor carpi radialis and abductor pollicis longus) and two muscles innervated by C8–T1 nerve roots but not by the median nerve (e.g., the first dorsal interosseous muscle and extensor indicis) were tested. The sternocleidomastoid muscle was examined when necessary. Spontaneous potentials at rest, the characteristics of motor unit potentials during mild contraction and recruitment potentials during maximal voluntary contraction were recorded and analyzed in all patients.

To grade the electrophysiological severity of CTS (17, 18), normal nerve conduction values from the EMG Laboratory of Peking Union Medical College Hospital were used as references, and a nerve conduction value that deviated from the mean by ±2.5 standard deviations was deemed abnormal. Severity grade was classified as mild, moderate or severe, with mild indicating a prolonged sensory nerve latency, a slightly prolonged DML (≤4.5 ms), normal SNAP and CMAP amplitudes (i.e., within the reference range) and normal findings on needle EMG; moderate denoting prolonged sensory nerve latency and DML (DML ≤ 6.0 ms), decreased CMAP and SNAP amplitudes (by up to 50%, if present) and mild abnormalities on needle EMG (e.g., a small number of spontaneous potentials); and severe indicating prolonged or unmeasurable sensory nerve latency and DML (DML > 6.0 ms), decreased SNAP and CMAP amplitudes (by >50%) or unmeasurable potentials, and the presence of spontaneous potentials or large, abnormal motor unit potentials during mild contraction on needle EMG. Patients were assigned to a mild, moderate or severe group based on the above grading criteria.

### 2.3 Data collection

This study comprises two parts; the first focuses on identifying anthropometric indicators associated with disease severity in patients with CTS, with the collected data including the following: (1) demographic data (sex, age, dominant hand and disease duration), (2) medical histories (history of pregnancy, endocrine and metabolic system diseases, cervical spondylosis, hand trauma or surgery, long-term use of neurotoxic drugs [e.g. platinum-based chemotherapeutic agents] and heavy alcohol consumption), (3) anthropometric measurements (height, body weight, body mass index [BMI], transverse carpal diameter and wrist circumference; all measurements were obtained using a standardized flexible measuring tape, except for transverse carpal diameter, which was measured with a digital caliper to enhance precision, and the measurement results are presented in millimeters [mm] and rounded to two decimal places) and (4) the results of EMG examinations of both upper extremities before treatment, measuring the distal sensory latency and DML of the median nerve, amplitude, nerve conduction velocity and other relevant electrophysiological parameters.

The second part of the study investigates the association between preoperative CTS severity and postoperative prognostic outcomes, with the scores of three questionnaires – the Levine Carpal Tunnel Questionnaire (LCTQ), the Patient-Rated Wrist Evaluation (PRWE) and the Disabilities of the Arm, Shoulder and Hand (DASH) questionnaire – collected at 3 weeks after treatment.

All evaluations and physical examinations were conducted by two attending physicians or senior specialists from the departments of neurology or orthopedics. Transverse carpal diameter was defined as the maximal distance between the most prominent points of the radial styloid process and the ulnar styloid process, measured on the dorsal aspect of the wrist with the hand in a neutral position. Wrist circumference was measured as the minimal circumference around the wrist, typically at the level of the distal wrist crease, with the wrist in a neutral, relaxed position. All measurements were performed by two experienced clinicians using a standardized rigid digital caliper (for diameter) or a flexible measuring tape (for circumference), and the mean of three consecutive measurements was recorded to ensure reliability. Height was measured as the maximum distance from the vertex of the head to the plantar surface of the foot, with the patient in an upright posture and the occiput, shoulders and buttocks maintained on a vertical plane. Body weight was recorded without shoes, socks or heavy clothing (19). For patients with unilateral involvement, transverse carpal diameter and wrist circumference were collected from the affected side; for those with bilateral involvement, measurements were taken from the side with subjectively more severe symptoms, and if the subjective severity was identical on both sides, the measurements from the dominant hand were used.

The scores from the LCTQ (20) were collected at 3 weeks after treatment. The LCTQ was designed to assess symptom severity in patients with CTS and covers multiple dimensions, including pain, paraesthesia, numbness, weakness, nocturnal symptoms and overall wrist function. These rigorous procedures ensured the accuracy of the questionnaire results, laying a solid foundation for subsequent analysis and discussion. The score for each individual item ranged from 1 to 5 points, with a lower total score indicating milder symptoms. The final total score was calculated as the sum of all individual item scores divided by the total number of items, with an intraclass correlation coefficient (ICC) of 0.91–0.93 and a Cronbach’s *α* coefficient of 0.89–0.91.

The DASH questionnaire consists of three sections (A, B and C), among which Section C is specifically designed for the assessment of patients with special occupations, such as pianists and athletes. Thus, only sections A and B of the DASH questionnaire were adopted in this study. Section A evaluates upper extremity function in daily living and social activities. Scores are assigned based on the difficulty level reported by patients, with 1 point indicating no difficulty, 2 points indicating mild difficulty, 3 points denoting moderate difficulty (with successful task completion), 4 points indicating severe difficulty and 5 points denoting an inability to complete the task. Section B assesses symptoms of discomfort in the upper extremity. Scores are rated according to symptom severity, with 1 point meaning no symptoms, 2 points indicating mild symptoms, 3 points indicating moderate symptoms, 4 points indicating severe symptoms and 5 points meaning extreme symptoms. The DASH Section A score was calculated as (total score/23–1) × 25, the DASH Section B score was calculated as (total score/7–1) × 25 and the total DASH score was calculated as [(total score of A + total score of B)/30–1] × 25. The DASH score ranges from 0 to 100 points, with a higher score indicating more severe upper extremity dysfunction and a lower score reflecting milder dysfunction. The questionnaire has a Cronbach’s *α* coefficient of 0.87–0.97, an ICC of 0.745–0.994 and a content validity index of 0.80–1.00 (21).

The PRWE comprises a total of 15 items: 5 pain-related items (4 assessing pain intensity and 1 evaluating pain frequency), 6 items related to specific activities and 4 related to difficulties partaking in daily activities of life, including self-care, household chores, work and recreation. Each item is assigned a score from 0 to 10, with a total score of 100 points for activity- and function-related items and 50 points for pain-related items. To obtain the total final score, the sum of the scores for the 10 activity- and function-related items is divided by 2 (maximum score: 50) and added to the total score of the pain-related items, resulting in an overall score ranging from 0 to 100 points. A higher score indicates more severe pain and upper extremity dysfunction (22). The PRWE has a Cronbach’s *α* coefficient of 0.87–0.97 and an ICC of 0.88–0.92.

### 2.4 Statistical analysis

Statistical analyses were performed using the SPSS version 26.0 software package (IBM, Armonk, NY, United States). The Kolmogorov–Smirnov test was applied for normality verification. Measurement data with a normal distribution were expressed as mean ± standard deviation. One-way analysis of variance was used to compare the means among multiple groups, and the Student–Newman–Keuls test was adopted for pairwise comparisons. Enumeration data were presented as frequency (*n*) or percentage (%) and analyzed using the chi-square test. Since disease severity was graded as an ordinal dependent variable (mild, moderate or severe), ordinal logistic regression analysis was used for multivariate assessment. Collinearity was evaluated using variance inflation factors (VIFs), with a VIF < 10 indicating no multicollinearity. Spearman’s rank correlation was used to evaluate the relationship between ordinal severity and questionnaire scores. A two-tailed *p*-value <0.05 was considered statistically significant.

## 3 Results

### 3.1 Comparison of related data among patients with different disease severities

With increases in disease severity, disease duration (14.66 ± 5.59 vs. 27.35 ± 11.67 vs. 34.81 ± 11.22, *p* < 0.001) and transverse carpal diameter (36.68 ± 3.03 vs. 38.78 ± 2.76 vs. 40.92 ± 2.89, *p* < 0.001) increased gradually. Compared with patients with mild CTS, the ratio of wrist circumference to transverse carpal diameter was lower in patients with moderate or severe CTS (4.24 ± 0.39 vs. 4.07 ± 0.39 vs. 3.92 ± 0.38, *p* < 0.001) (see Table 1).

**Table 1:** Comparison of related data among patients with different disease severities.

Item	Mild grade (*n* = 46)	Moderate grade (*n* = 43)	Severe grade (*n* = 59)	*p*-value
Age (years)	48.96 ± 5.93	50.19 ± 7.32	49.42 ± 7.67	0.711
Sex (male/female)	9/37	8/35	10/49	0.940
Unilateral or bilateral involvement	6/40	6/37	7/52	0.951
Disease duration (months)	14.66 ± 5.59	27.35 ± 11.67^a^	34.81 ± 11.22^ab^	<0.001
BMI (kg/m^2^)	24.05 ± 2.45	23.73 ± 3.25	24.08 ± 2.87	0.806
Transverse carpal diameter (mm)	36.68 ± 3.03	38.78 ± 2.76 ^a^	40.92 ± 2.89^ab^	<0.001
Wrist circumference (mm)	154.82 ± 12.85	156.91 ± 12.53	160.00 ± 11.82	0.100
Wrist circumference/Transverse carpal diameter (mm)	4.24 ± 0.39	4.07 ± 0.39 ^a^	3.92 ± 0.38 ^a^	<0.001

BMI, Body Mass Index. ^a^Statistically significant difference compared with the mild group; ^b^Statistically significant difference compared with the moderate group.

### 3.2 Ordinal logistic regression analysis of factors associated with disease severity in patients with carpal tunnel syndrome

To further investigate the influencing factors of electrophysiological severity in patients with CTS, ordinal logistic regression analysis was performed, with CTS severity (ordinal variable: mild, moderate or severe) as the dependent variable and the variables with statistically significant differences in univariate analysis (disease duration, transverse carpal diameter and wrist circumference/transverse carpal diameter) as the independent variables. The omnibus test of model coefficients was statistically significant (*p* < 0.001). The Nagelkerke pseudo *R*^2^ was 0.622, indicating that the three independent variables accounted for 62.2% of the variance in CTS severity. No multicollinearity was detected (all VIF < 10). The results indicate that disease duration, transverse carpal diameter and wrist circumference/transverse carpal diameter are independent influencing factors for preoperative electrophysiological severity (all *p* < 0.001). The detailed results are presented in Table 2.

**Table 2:** Ordinal logistic regression analysis of factors associated with disease severity in patients with CTS.

Item	*β*	SE	OR	95%CI lower limit	95%CI upper limit	*P*-value	VIF
Disease duration	0.214	0.025	1.238	1.180	1.299	<0.001	0.903
Transverse carpal diameter	0.152	0.020	1.164	1.120	1.210	<0.001	0.560
Wrist circumference/Transverse carpal diameter	−0.511	0.127	0.599	0.469	0.766	<0.001	0.522

CTS, Carpal Tunnel Syndrome; Ordinal logistic regression model; Omnibus test *P* < 0.001; Nagelkerke pseudo *R*^2^ = 0.622. OR, odds ratio; CI, confidence interval; VIF, variance inflation factor.

### 3.3 Correlation analysis between disease severity and early postoperative hand function status in patients with carpal tunnel syndrome

To evaluate postoperative recovery across groups, group comparisons were performed for preoperative and postoperative LCTQ, PRWE and DASH scores. Postoperative scores at 3 weeks were significantly improved compared with baseline in all groups (all *p* < 0.001). Specifically, postoperative LCTQ (24.33 ± 6.45 vs. 26.12 ± 1.48 vs. 31.95 ± 2.65, *p* < 0.001), PRWE (45.57 ± 3.69 vs. 48.44 ± 2.33 vs. 51.05 ± 6.48, *p* < 0.001) and DASH (71.57 ± 3.80 vs. 77.88 ± 1.62 vs. 82.15 ± 3.32, *p* < 0.001) scores increased gradually with higher electrophysiological severity (all *p* < 0.001). All postoperative scores were significantly lower than baseline (*p* < 0.001), indicating objective early postoperative hand function status (see Table 3). Spearman’s rank correlation was performed separately to assess the linear relationship between preoperative electrophysiological severity and questionnaire scores. The results showed that preoperative electrophysiological severity was positively correlated with preoperative LCTQ (*r* = 0.63), PRWE (*r* = 0.42) and DASH (*r* = 0.87) scores (see Figure 1).

**Table 3:** Correlation analysis between preoperative disease severity and prognostic outcomes in patients with CTS.

Item	Mild grade (*n* = 46)	Moderate grade (*n* = 43)	Severe grade (*n* = 59)	*P*-value
Preoperative Levine score (points)	42.65 ± 7.12	48.92 ± 6.85	56.78 ± 7.34	<0.001
Postoperative Levine score (points)	24.33 ± 6.45	26.12 ± 1.48 a	31.95 ± 2.65 ab	<0.001
Levine score improvement (points)	18.32 ± 4.11	22.80 ± 5.26	24.83 ± 5.79	<0.001
Preoperative PRWE score (points)	68.42 ± 5.36	75.16 ± 6.22	82.35 ± 7.18	<0.001
Postoperative PRWE score (points)	45.57 ± 3.69	48.44 ± 2.33 a	51.05 ± 6.48 ab	<0.001
PRWE score improvement (points)	22.85 ± 3.84	26.72 ± 4.51	31.30 ± 5.22	<0.001
Preoperative DASH score (points)	89.66 ± 4.25	96.43 ± 5.18	102.52 ± 6.33	<0.001
Postoperative DASH score (points)	71.57 ± 3.80	77.88 ± 1.62 a	82.15 ± 3.32 ab	<0.001
DASH score improvement (points)	18.09 ± 3.16	18.55 ± 3.79	20.37 ± 4.15	<0.001

CTS, Carpal Tunnel Syndrome; PRWE, Patient-Rated Wrist Evaluation; DASH, Disabilities of the Arm, Shoulder and Hand. ^a^Statistically significant difference compared with the mild group; ^b^Statistically significant difference compared with the moderate group.

**Figure 1:**
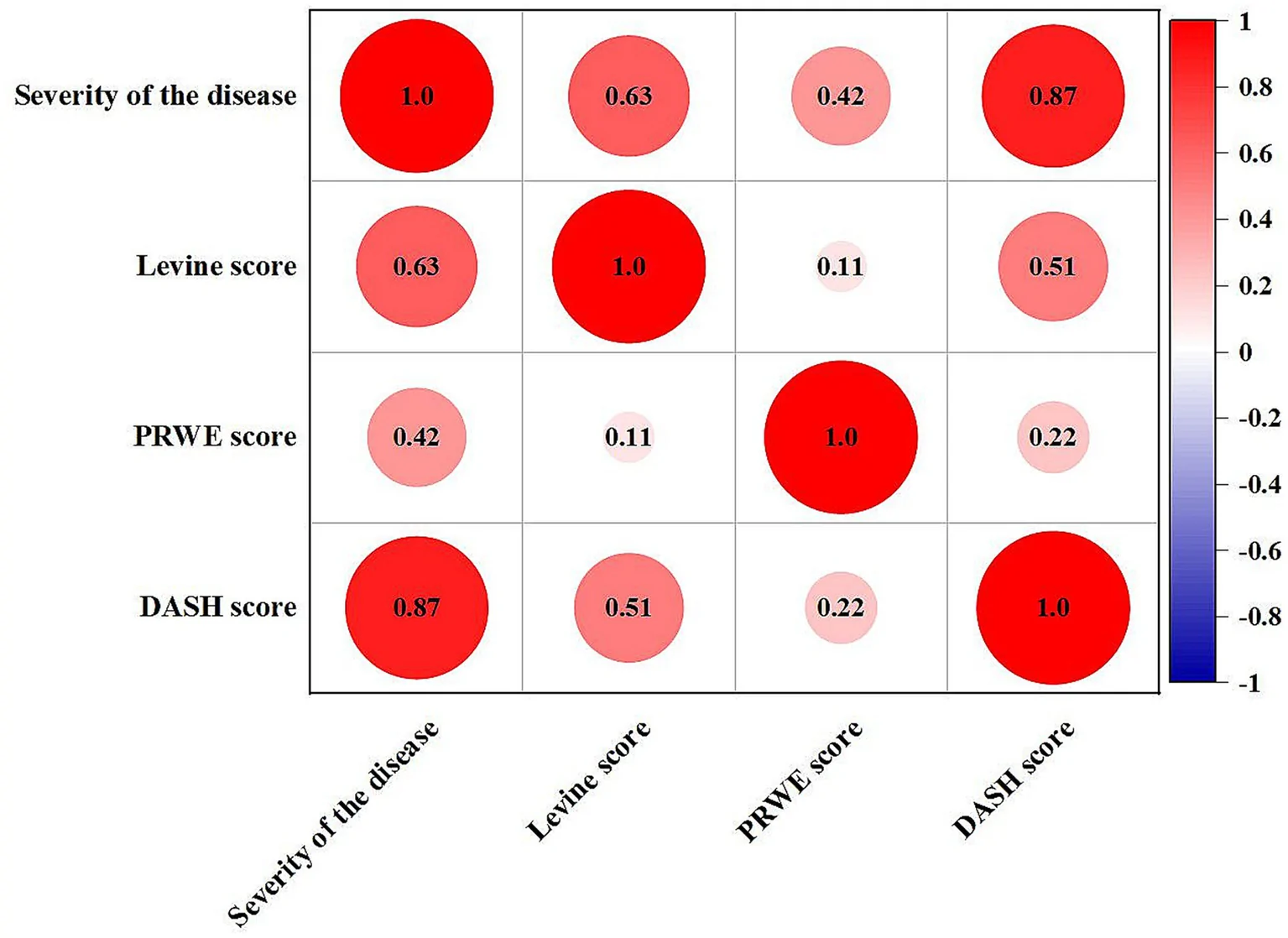
Correlation analysis among various indicators.

## 4 Discussion

Women accounted for 81.8% of the study population. This is consistent with the findings of relevant epidemiological investigations into CTS occurrence (23). However, Mondelli et al. (12) note that the incidence of CTS in men might be underestimated, as the proportion of women seeking medical care for CTS is higher than that of men. According to a epidemiological survey on CTS, the male-to-female ratio of patients with CTS is approximately 1:1.4 (24), whereas the ratio in this study is 1:4.48, and selection bias among participants cannot be excluded.

A large body of research has confirmed that female gender is a risk factor for CTS onset, with the proposed underlying reasons including the generally smaller carpal tunnel in women and their greater involvement in household chores. Fujimaki (25) analyzed the effects of age, sex, BMI and other indicators on median nerve SNAP and found that the median nerve SNAP in women was significantly higher than that in men (*β* = −6.519, *p* = 0.017), suggesting that women may experience a higher degree of electrophysiological severity than men. However, the data from this study show no significant difference in the electrophysiological severity of CTS between genders (*p* = 0.940). Kim et al. (26) provided additional evidence that female gender is a protective factor in CTS. Retrospectively analyzing the impact of hormone replacement therapy on NCSs in perimenopausal women, they found that the results of both motor and sensory NCSs were significantly superior in an estrogen treatment group than in an untreated group. This suggests that estrogen may act as a protective factor for peripheral nerves (27). The underlying mechanism for this may be that estrogen receptors are distributed on the surface of peripheral nerves and can regulate nerve proliferation and regeneration (28). At present, there are still relatively few studies focusing on the electrophysiological severity of CTS. Only Mondelli et al. (12) integrated clinical manifestations with electrophysiological severity in their research, with the conclusion that gender is not associated with CTS severity. This is consistent with the findings of the current study and indicates that, in clinical practice, gender should not be used as a determining factor of CTS severity in patients.

Several studies have indicated that disease duration is positively correlated with sensory conduction latency and motor conduction latency, yet cases with unelicited latencies were excluded in these studies – a detail that could lead to selection bias within the respective study populations (29). The present study identified disease duration as an independent risk factor for electrophysiological severity in CTS. Although previous studies have evaluated the relationship between disease duration and clinical severity/postoperative prognosis in CTS, relatively few studies have specifically focused on the direct association between disease duration and electrophysiological severity using standardized grading criteria. Only a few studies have noted an association between disease duration and CTS prognosis, suggesting that a longer disease duration may be associated with a poorer prognosis (26). This implies that clinicians should exercise caution when assessing CTS severity in patients with a long disease duration and adopt more aggressive treatment strategies.

Current research has identified median nerve transverse diameter as a potentially useful measure for diagnosing patients with CTS and predicting the condition’s severity (11) – a finding consistent with the results of the present study. Furthermore, the findings of this study demonstrate that wrist circumference is not an influencing factor for the electrophysiological severity of CTS. This may be attributable to the fact that wrist circumference is correlated with the longitudinal and transverse diameters of the wrist (i.e., transverse carpal diameter) and is affected by such factors as osseous structures (including the radius and ulna) and fat distribution in the wrist, making it an unsuitable indicator for assessing CTS severity. Given that neither transverse carpal diameter nor wrist circumference can objectively reflect fat distribution in the wrist, the ratio of wrist circumference to transverse carpal diameter may serve as a more optimal indicator of severity, with studies reporting that a smaller ratio is associated with a longer DML in NCS examinations of patients with CTS (30). Mondelli et al. (12) further confirmed a negative correlation between the wrist circumference-to-transverse carpal diameter ratio and CTS severity, identifying this ratio as the most accurate predictor of CTS severity. The present study reached a similar conclusion; however, no statistically significant difference in this ratio was observed between patients with moderate and severe CTS. We speculate this is due to the study’s relatively small sample size.

In the present study, three questionnaires – the LCTQ, DASH and PRWE – were employed to assess the postoperative prognostic outcomes of patients. These three questionnaires differ in their research focuses (20–22). The LCTQ is specifically designed for CTS and primarily captures the symptoms caused by median nerve compression (e.g., numbness and pain) and its associated functional limitations (e.g., difficulty in grasping). The DASH questionnaire assesses the overall motor function of the upper extremity, including the motor capacity and symptoms (e.g., pain and numbness) of the shoulder, elbow, wrist and hand, with an emphasis on the correlation between patients’ subjective experiences and difficulty in completing daily activities. The PRWE focuses on wrist joint function, evaluating pain intensity and the degree of limitation patients experience during specific activities (e.g., lifting objects and twisting bottle caps). It is suitable for the short-term monitoring of function in local wrist injuries and chronic wrist disorders and is particularly applicable to clinical scenarios requiring rapid assessment of the functional status of the wrist joint. These three questionnaires in conjunction enable a more comprehensive reflection of patients’ prognostic outcomes. In this study, a higher preoperative electrophysiological severity was associated with a poorer postoperative function status. Although this is consistent with the findings of relevant studies (30), there is still a lack of studies directly investigating the association between preoperative electrophysiological severity and the scores of these three questionnaires. Moreover, it is important to acknowledge that the 3-week postoperative timepoint assessed in this study represents a very early phase of hand function status. At this stage, improvements in LCTQ, PRWE and DASH scores are likely attributable to early symptom relief (e.g., a reduction of nocturnal paraesthesia) and wound healing rather than definitive axonal regeneration or true neurological status.

The present study has certain limitations that should be acknowledged. It is a single-center, retrospective clinical study with a relatively small sample size. The study population included only patients with positive EMG findings, with relevant data lacking for those with negative EMG results. In addition, patients with mild-to-moderate CTS may not have sought medical care. Thus, the study fails to fully reflect the actual clinical spectrum of CTS and is subject to selection bias. Future research should expand the sample size and conduct prospective investigations. The short follow-up duration of 3 weeks is also a notable limitation. This early timepoint does not allow for the assessment of sustained neurological improvement or the prevention of long-term sequelae such as muscle atrophy. Future studies with extended follow-up periods (e.g., 6 or 12 months) are required to validate the long-term prognostic value of preoperative electrophysiological severity. Furthermore, the LCTQ is a disease-specific instrument for evaluating CTS, whereas the PRWE and DASH questionnaires assess general upper limb function. It is important to acknowledge that these broader measures may be influenced by comorbidities or conditions outside the carpal tunnel (e.g., shoulder and elbow pathologies), and this could affect their sensitivity in detecting CTS-specific recovery. Therefore, the findings related to PRWE and DASH scores should be interpreted with caution, as they may not exclusively reflect the effects of therapeutic interventions on CTS.

## 5 Conclusion

Disease duration, transverse carpal diameter and the ratio of wrist circumference to transverse carpal diameter are correlated with electrophysiological severity in patients with CTS. Longer disease duration, larger transverse carpal diameters and smaller wrist circumference-to-transverse carpal diameter ratios are associated with a higher degree of electrophysiological severity and a poorer early postoperative function status in these patients.

## Data Availability

The original contributions presented in the study are included in the article/supplementary material, further inquiries can be directed to the corresponding author.
